# Tourism Development During the Pandemic of Coronavirus (COVID-19): Evidence From Iran

**DOI:** 10.3389/fpubh.2022.881381

**Published:** 2022-03-29

**Authors:** Zeynab Hallaj, Masoud Bijani, Enayat Abbasi, Naser Valizadeh, Maryam Mohammadi

**Affiliations:** ^1^Department of Agricultural Extension and Education, College of Agriculture, Tarbiat Modares University, Tehran, Iran; ^2^Department of Agricultural Extension and Education, School of Agriculture, Shiraz University, Shiraz, Iran

**Keywords:** tourism, coronavirus, social exchange theory (SET), causal analysis, Sistan

## Abstract

The coronavirus (COVID-19) epidemic has created a great deal of fear and uncertainty about health, economy, and social life. Therefore, the health, social, and economic impacts of COVID-19 are of great importance. In prone rural communities, tourism industry can contribute to the sustainable economy and social development of the villagers, and as a dynamic economic sector, cause economic, social, cultural, and environmental changes. In this regard, the purpose of this inquiry was to develop tourism during the coronavirus pandemic using the social exchange theory (SET). The present study is a descriptive, correlational and causal inquiry that is conducted using survey technique. The statistical population included tourists visiting Sistan region around Hamoun Wetland in eastern Iran (*N* = 850). In the sampling process, 266 tourists were selected as a sample using random sampling strategy. The study instrument was a researcher-made questionnaire, whose validity was confirmed by a panel of subjectivists and its reliability was approved by a pilot study and Cronbach's alpha coefficients (0.87≥ α ≥ 0.71). Based on SET, the proposed causal model was able to explain about 56% (${\text{R}}_{\text{Adj}}^{2}$ = 0.562) of the variance changes in tourism development during the COVID-19 epidemic.

## Introduction

Today, tourism is a phenomena in terms of income generation, which has been emphasized in many countries of the world and a lot of investment is made in this sector (1). It is an industrial tourism that has attracted the attention of many tourists (2). In this regard, geographical, topography, climate and location are the most important elements for tourist attractions that play an important role in economic growth and social development of many regions (3). Iran is one of the largest countries in the Middle East and has its origins in the ancient Zoroastrian religion (4). With a rich heritage, it includes 13 cultural sites in the UNESCO World Heritage List (5), and has a variety of climates, seasons, lakes, plains, caves, and deserts (6) that have led to the expansion of tourism in the country. Sistan and Baluchestan province with an area of about 180,726 square kilometers, after Kerman province, is the second largest province in Iran. Sistan region is located in the north of this province, which comes from the alluvium of Helmand River, which is the largest freshwater lake in the world in times of water. Khajeh mountain is the only high ridge that is located in Sistan and has a special sanctity among the people. Hamoun River of Sistan and its half wells are among the water resources of this region. In June 2015, Kalpurgan pottery, Sistan embroidery, and Baluchistan needlework have been awarded UNESCO. Sistan tourist areas, including Shahr-e Sokhteh, Hamoun lake, Khajeh mountain and infidels' castle, Bibi Doust, Chehel Dokhtar castle, throne of justice, half well, three mountains, Ramroud Castle, Sam Castle, Rostam Castle, Malek Kiani Khan Citadel, Chehel Dokhtaran, British Embassy, Sardar Mohammad Hussein Citadel, etc. are among the natural and historical tourist attractions of Sistan (7). However, although the tourism industry is growing rapidly, it is not safe from global health emergencies, diplomatic warfare, terrorism, and natural disasters. The outbreak of coronavirus is not the first case to occur in the 21st century (8). Tourism is one of the sectors that has suffered the most from governments following this epidemic. The epidemic led to restrictions on movement and travel (9). The tourism economy has been severely affected by the COVID-19 epidemic, which has caused unprecedented damage to the tourism sector. Measures have been taken to curb its expansion, including lifting travel restrictions, restoring traveler confidence, and reviewing the tourism sector for the future (10). Community suspensions, social distance, home stay orders, travel and mobility restrictions have led to the temporary closure of many tourism-related businesses and a significant reduction in demand for businesses that were allowed to continue operating (11). Restrictions imposed by governments have led to drastic reductions in employment and incomes (10), and these barriers play a significant role in tourism growth (12).

Rural areas have a good capacity to develop tourism activities due to their location and important elements to attract tourists, such as natural landscapes, antiquities, climatic diversity, social customs and traditions (13) and it can be said that the promotion of non-agricultural activities, such as tourism development, can be an effective factor in increasing the welfare of low-income rural families, strengthening the economic base of centers, and reducing migration to cities (14, 15). Tourism study has focused primarily on residents' perceptions, recent studies have focused their efforts on analyzing tourists' perceptions of the effects and promotion of tourism on a destination (16, 17). In addition, researchers have argued that tourists are aware of the importance of tourism impact and tourism development in a destination, especially of the interests of the community or the protection of tourism or environmental awareness and conservation behavior (16–18). Residents of the host community are mainly and directly affected by tourism development (19). At the same time, they play an important role in the quality of tourists' experiences in the community (20). Understanding the relationship between residents and tourists, from the perspective of local residents, is a key element in the sustainable tourism destinations (21).

According to what was stated, the purpose of this research was to investigate the development of tourism during the corona virus epidemic in Sistan, Iran. In this regard, the novelty of this research should be considered from two aspects. First, the place of the research was the pristine bedrock of the subject. The second was its epistemological perspective in the form of a theoretical framework derived from social exchange theory (SET).

## Theoretical Background

Sustainable behavior is an emerging challenge in tourism (22, 23) and can play a key role in meeting the various challenges facing rural areas (24). It also leads to the revitalization of rural areas through protecting the quality of life of residents, preserving traditional cultures, protecting environment, creating employment, and generating income (25).

Social Exchange Theory (SET) is one of the most influential conceptual paradigms and one of the oldest theories for analyzing social behavior and the link between the disciplines of anthropology, social psychology and sociology. It includes interactions that create commitments (26). SET is one of the oldest theories of social behavior (27) that depends on the ability to create high quality relationships under certain conditions (in this study, specific conditions are pandemic of COVID-19) (28) and explains any kind of interaction between people and resources (29). The resources that are exchanged may be not only tangible, but also intangible, such as social facilities or friendship (29). The basic premise is that parties enter into a relationship and maintain their relationship with the expectation that doing so will be rewarding (27, 30). For example, in this research based on SET, if more benefits and positive effects are understood, tourists will be more supported and have interaction (31). Tourists' perception toward tourism and relationship between tourists and hosts are identified as key factors in the tourism and important issues for governments, policymakers, and industry (32). From a psychological perspective, attitudes are specifically defined as a tendency to evaluate individuals, issues, and more (33). One of the advantages of using SET is that it can explain perceptions as well as examine residents' relationships both individually and collectively (34).

**Figure 1:**
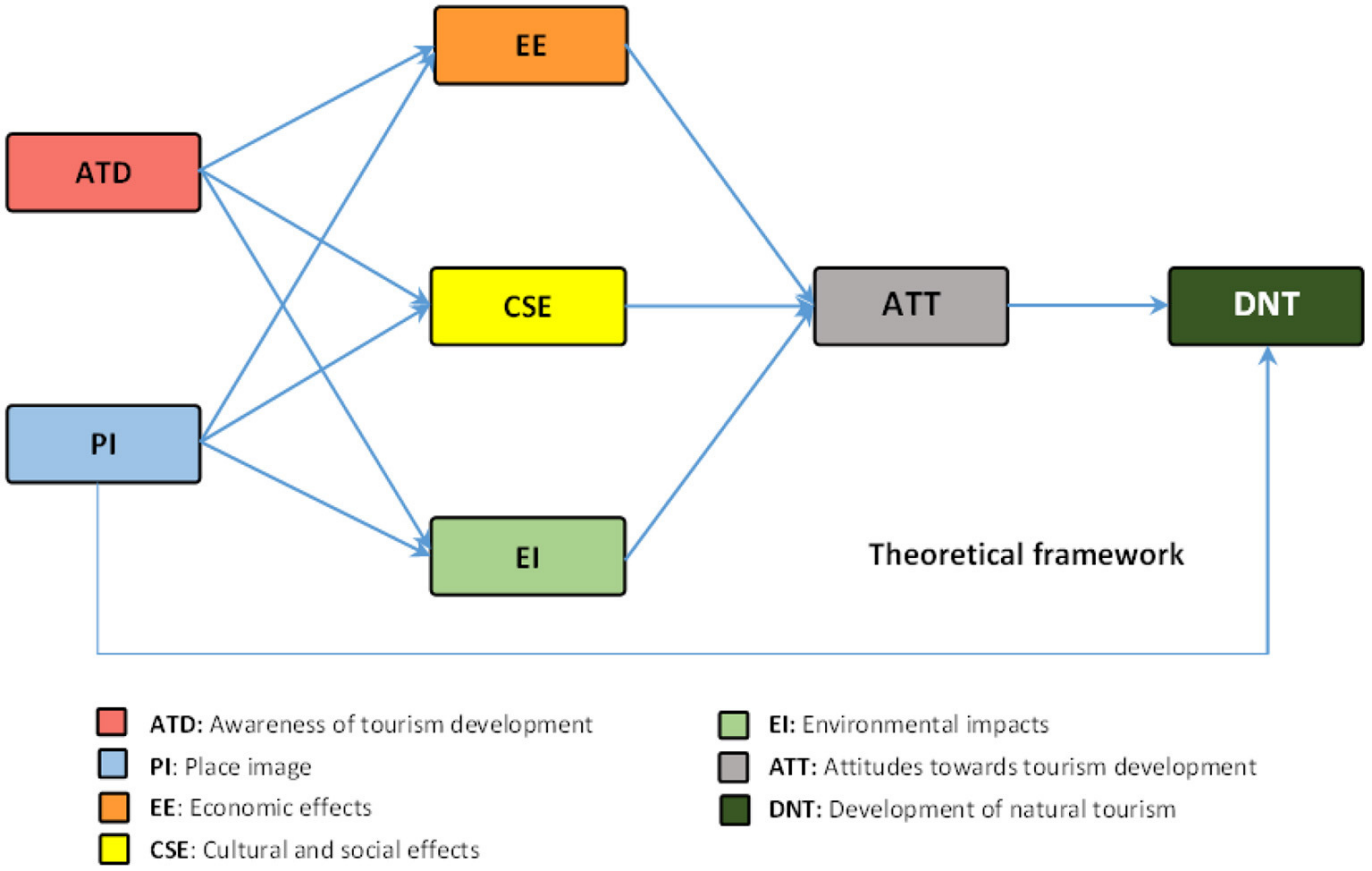
Theoretical framework of the study based on SET in the analysis of tourism development in the pandemic of COVID-19 (44).

Impacts of tourism development are divided into three categories: economic, environmental and social (35). Among the key elements in sustainable tourism are environmental conservation, strengthening communities, and preserving traditional cultural heritage (36) by making changes in moral and social norms and values (37, 38). It is usually associated with a positive impact on the services provided by the community to its residents (39), which allows the creation of new recreational opportunities (40). In particular, creating job opportunities and increasing household income for locals is economically important (41). In fact, economic results are those that are tangible and address financial needs. Socio-cultural consequences are those that address an individual's social needs and self-esteem and are often symbolic and specific (28). On the other hand, tourism has a great deal of responsibility for environmental activities. It is destructive to the environment if these activities are not carried out in the right places and the necessary measures are not taken to prevent damage to the environment (42). Economic, social and environmental effects have a reinforcing effect on the conservation of natural resources and the improvement of various aspects related to the environment (39). From a tourism perspective, SET suggests that people's attitudes toward tourism (ATT) and the consequent level of support for its development will be influenced by their assessments of the consequences of tourism for themselves and their communities (43). Another factor related to understanding the reaction to tourism is the image of the place. If tourism development is to the benefit of the local community, it should also focus on residents' perceptions of places and tourists, which is a relatively stable psychological trait (44). In this regard, Figure 1 is presented as a theoretical framework of this study.

Some of the studies carried out in this regard are mentioned below.

Stylidis et al. examined the role of residents' place image (PI) in shaping their support for tourism development. The tested model suggested that residents' PI affects their perception of tourism impacts and in turn their support for tourism development. The results emphasized the need for a more flexible, resident-based measure of tourism impacts and showed that a better understanding of the economic, socio-cultural, and environmental impacts leads to greater support. In addition, while tourism development studies have largely ignored residents' image of the location, the findings of this study showed its importance in shaping residents' perceptions of tourism impacts as well as their level of support (45).

Ghazani et al. stated that the villagers believe that promotional education in the field of tourism leads to increased interaction, making more money, not evacuating the village, the prosperity of rural industries such as tourism, agricultural stabilization, and preservation of the rural environment (46).

Hallaj and Karimi Goghari emphasized the need to attract tourists and considered it as a factor to increase the income of villagers, which reduces their migration by creating more jobs for villagers. He states that by encouraging the villagers to preserve historical attractions and natural landscapes and educating them and encouraging them to preserve local customs and traditions in the face of cultural changes and reviving the original and indigenous culture of the village, attracting tourists will flourish (47).

Ghazani et al. emphasized the identification of bottlenecks and taking action to eliminate them for the prosperity of natural tourism. He stated that providing infrastructure facilities such as tourist stations, roadside resorts with suitable amenities, increasing the number of guest houses with local structure and training of local specialists, and increasing the awareness of villagers, the development of tourism can be supported (48).

Lee and Jan examined community-based tourism and its role in sustainable development, emphasizing the sustainability of nature-based tourism. He stated that economic, socio-cultural and environmental sustainability significantly change in the stages of stabilization, development and participation of community-based tourism development (49).

Streimikiene et al. (2021) examined the main forms and factors of strengthening tourism competition by implementing economic, social and environmental goals of tourism destination development. They stated that participants of businesses are interested in new technologies in tourism services that have a positive impact on the environment and local communities (50).

Qin et al. examined the support of residents using SET. They stated that residents' support for tourism is influenced by perceived personal interests and the positive effects of tourism. Residents' tolerance for tourism plays an important moderating role in the relationship between perceived negative tourism effects and support for tourism development. Residents with less tolerance for tourism were more sensitive to the negative effects of tourism, and therefore, tended to express less support for tourism development (51).

Chang examined the effective attitudes on tourism development based on SET and social network theory. They showed that social network (intelligence, friendship, and counseling) plays different roles in the three components of tourism development attitude (cognition, interest, and pragmatism). Integrating the evaluation of the value of profit of SET and the orientation perspective of the relationship between social network theory and understanding the impact of social network relationship links on tourism development attitudes is effective. Intelligence and friendship have a positive and significant effect on tourism development hierarchy (52).

Çelik and Rasoolimanesh used the theory of rational action (TRA) and SET and showed that negative and positive attitudes of residents have a direct effect on tourism-cost-benefit attitudes and indirect effects on tourism support. Also, the cost-benefit attitude plays a mediating role between residents' attitudes toward tourism and its support (53).

Nagaj and Žuromskaite stated that this disease not only affects the health system and people's health, but also the economy. The effects of this disease include restrictions on movement and travel, which have affected both domestic and international tourism; however, this epidemic has reduced greenhouse gas emissions in tourist areas (9).

Samdin et al. addressed the main concerns of tourists in deciding on a destination and travel risk that could affect their safety. They stated that priorities such as safety and health information are the strongest predictors of tourists' decision-making, and the media also play an important role in raising awareness for tourism development (8).

Budayana and Adi stated that in areas that rely on rural tourism, tourism during the epidemic cannot be stopped; because the economics of these societies depend on tourism. In this case, incentives should be provided to innovate to improve rural tourism in rural areas and improve local trade (54).

Based on what has been stated so far and based on the theoretical framework presented in Figure 1, the following hypotheses have been developed. In this regard, it can be assumed that there is a significant relationship between tourism development and:

- tourism facilities,
- job creation,
- tourists' attitudes,
- tourist awareness,
- its socio-cultural effects,
- its economic effects,
- the resulting environmental impact and
- tourists' image of location.

## Research Methodology

**Figure 2:**
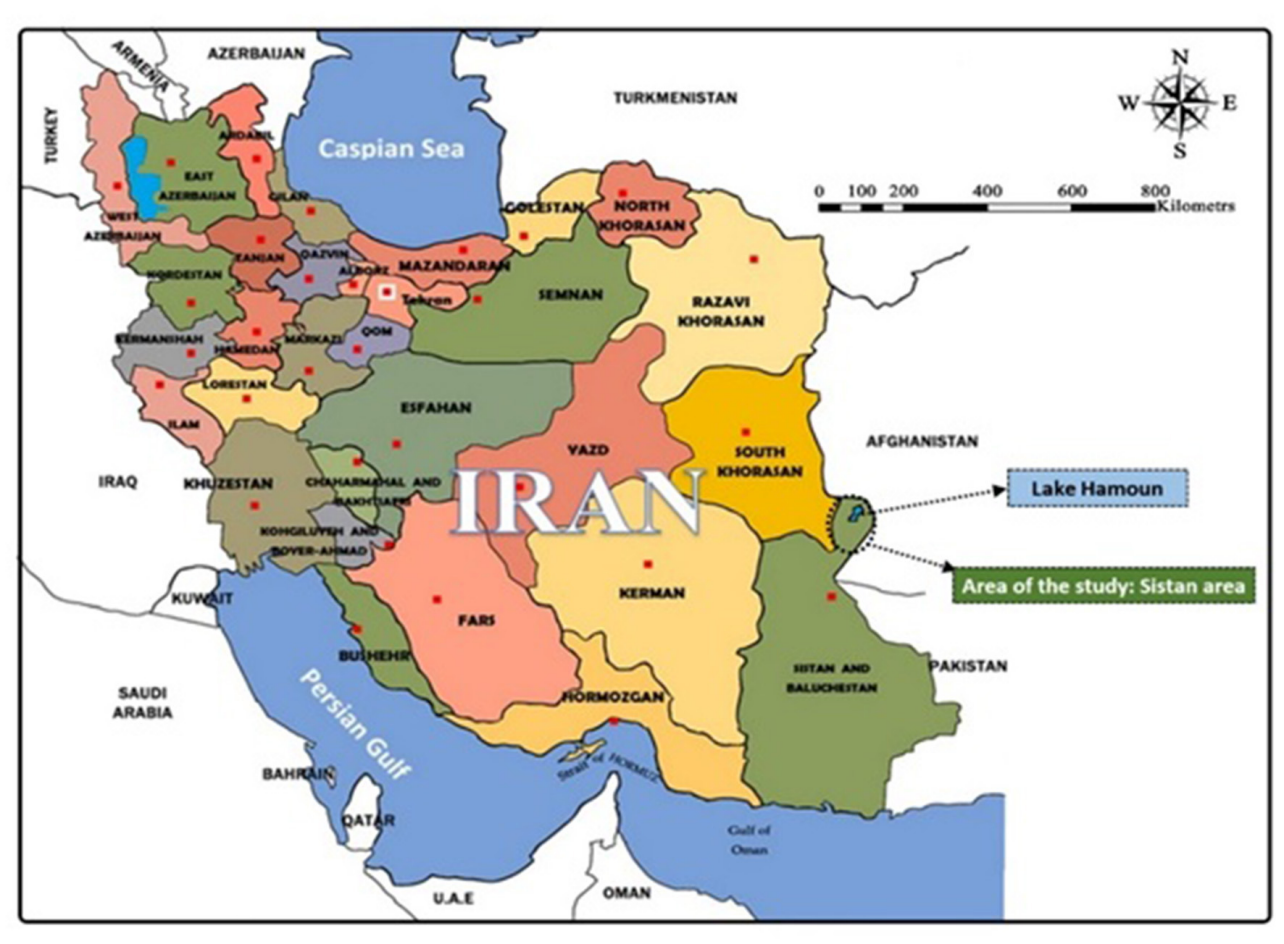
The location map of Sistan area in Iran.

The present study is an applied research. The study population included tourists in Sistan region in 2020–2021 (*N* = 850) and the sample size was estimated at 266 people based on Krejcie and Morgan sampling table (55). A simple random method was used for sampling. The research tool in this study was an electronic closed-ended questionnaire whose validity was confirmed using the opinions of experts. The reliability of the questionnaire was calculated among tourists of Khash city (outside the statistical population but similar to it) and using Cronbach's alpha test for variables measured by Likert scale, turning out to be at an acceptable level (Table 1). The location of the study was in the Sistan region in the catchment area of Hamoun Lake, eastern Iran (Figure 2).

**Table 1:** Items of research variables and Cronbach's alpha coefficients.

**Variables**	**No**.	**Items**	**References**
ATD	**Awareness of tourism development (ATD): (α** **=** **0.77)**		
	1	I have information about the area I am entering during COVID-19.	(34)
	2	I am aware of the government's managerial role in the development of tourism during COVID-19.	
	3	I have a lot of economic information about tourism development during COVID-19.	
	4	I have a lot of cultural information about the development of tourism during COVID-19.	
PI	**Place image (PI): (α** **=** **0.83)**		
	1	The people of Sistan are reliable.	
	2	In case of emergency for me, the people of Sistan will be eager to help me.	(45)
	3	The environment of this region is beautiful to me even in the face of drought.	
	4	In Sistan region, there is a sincere and intimate relationship between tourists and residents of the region.	
	5	I feel safe even when I travel alone in the area.	
	6	Tourism officials do their job well.	
CSE	**Cultural and social effects (CSE): (α** **=** **0.73)**		
	1	The quality of public services has improved in the region because of COVID-19.	(56, 57)
	2	With the development of tourism during COVID-19, more recreational opportunities will be available to tourists and locals.	
	3	There are many shopping opportunities in this area for tourists, even during COVID-19 conditions.	
EI	**Environmental impacts (EI): (α** **=** **0.87)**		
	1	Tourism during COVID-19 and the presence of tourists have a negative impact on the environment of the region.	(56, 57)
	2	Tourist areas (Khajeh Mountain and Shahr-e Sokhteh, etc.) become very crowded due to the presence of tourists, even during COVID-19.	
	3	Long-term planning by tourism officials in the region can control the negative effects of tourism and COVID-19.	
EE	**Economic effects (EE): (α** **=** **0.75)**		
	1	The tourism industry plays an important role in the economy of the Sistan region during COVID-19.	(57)
	2	In my opinion, the economic benefits of tourism during COVID-19 are greater than the negative consequences.	
	3	Tourism during COVID-19 in this area has improved the quality of life of the people.	
	4	The development of tourism in the region during COVID-19 could create more job opportunities for local people.	
DNT	**Development of natural tourism (DNT): (α** **=** **0.84)**		
	1	In my opinion, the development of tourism during COVID-19 should continue actively in this region.	(45, 58), self-administrated
	2	I support tourism during COVID-19 and I would like to see tourism flourish in this area.	
	3	In my opinion, the Sistan region should be recognized as a tourist destination.	
	4	I fully support the development of tourism during COVID-19 in the Sistan region.	
ATT	**Attitudes toward tourism development (ATT): (α** **=** **0.71)**		
	1	Tourism during COVID-19 around Sistan region should be based on the use and protection of nature and culture of the region.	
	2	Tourism in the lagoon should not disrupt the habitat of plants and animals in the area.	(59), Self-administrated
	3	Tourism development in the region during COVID-19 should focus on environmental education.	
	4	Tourism in the region during COVID-19 promotes environmental awareness among tourists.	
	5	Tourism in the lagoon during COVID-19 should be done in small groups.	
	6	Tourism in the lagoon should be limited during important time periods (bird mating season, etc.).	

The main dependent variable in the present study is tourism development and in accordance with the presented theoretical framework (Figure 1), the independent variables affecting it were awareness, PI, economic, social, cultural and environmental effects and attitudes toward tourism (ATT). For to measuring the variables, five-point Likert scale was used [strongly disagree (1), disagree (2), undecided (3), agree (4), and strongly agree (5)]. To measure each of the variables, items were extracted from previous related studies. In the absence of a suitable item for measuring the variables, researcher-made items that have been approved by experts were used.

## Results and Discussion

### Descriptive Statistics of the Respondents' Individual and Professional Characteristics

The average age of the respondents was ~30.17 years and most of them (52.6%) were in the age range of 30 years. Also, 66.5% of them were men and the rest were women. About 46% of them had a bachelor's degree or higher, 62.8% of them traveled to Sistan more than once, and 93.6% of them traveled to this region during the drought. Also, Khajeh Mountain with 28.4%, Shahr-e Sokhteh with 22.3%, and Qale-e-No village with 15.5% had the highest visit statistics (Table 2).

**Table 2:** Descriptive statistics of individual and professional characteristics.

**Variables**	**Levels**	**Frequency**	**Percent**	**Cumulative percent**	**Mean**
Age (years)	Young: X_i_ <29	140	52.6	52.6	30.17
	Middle-aged: 29 ≤ X_i_ <43	101	38	90.6	
	Elderly: 43 ≤ X_i_	25	9.4	100	
Gender	Female	89	33.5		
	Male	177	66.5		
Education level	Illiterate	1	0.4	0.4	
	Primary education	1	0.4	0.8	
	Middle literacy	6	2.3	3.0	
	High school to diploma	68	25.6	28.6	
	Associate degree	66	24.8	53.4	
	Bachelor's degree	84	31.6	85.0	
	Master degree	36	13.5	98.5	
	Ph.D.	3	1.1	99.6	
	Theological	1	0.4	100	
Travel history	Yes	167	62.8		
	No	99	37.2		
Travel time	Drought	249	93.6		
	Wet year	17	6.4		
Income (US dollars per year)	Low: X_i_ <680	254	95.5	95.5	
	Medium: 680 ≤ X_i_ <1,360	9	3.4	98.9	
	High: 1,360 ≤ X_i_	3	1.1	100	

### Correlation Analysis Between Variables

For examining the relationships between variables, Pearson correlation coefficient was used (see Table 3). The results reveled that there was a significant positive correlation between PI and ATD (*p* < 0.01; *r* = 0.419). Other variables, including CSE (*p* < 0.01; *r* = 0.314), EI (*p* < 0.01; *r* = 0.145), EE (*p* < 0.01; *r* = 0.221), DNT (*p* < 0.01; *r* = 0.293), and ATT (*p* < 0.01; *r* = 0.248), also had a positive and significant correlation with ATD. Also, the variables CSE (*p* < 0.01; *r* = 0.454), EI (*p* < 0.01; *r* = 0.286), EE (*p* < 0.01; *r* = 0.453), DNT (*p* < 0.01; *r* = 0.528), and ATT (*p* < 0.01; *r* = 0.391), showed positive and significant relationships with PI.

**Table 3:** The variables correlation matrix according to the theoretical framework.

	**ATD**	**PI**	**CSE**	**EI**	**EE**	**DNT**	**ATT**
ATD	1						
PI	0.419^**^	1					
CSE	0.314^**^	0.454^**^	1				
EI	0.145^**^	0.286^**^	0.377^**^	1			
EE	0.221^**^	0.453^**^	0.369^**^	0.234^**^	1		
DNT	0.293^**^	0.528^**^	0.402^**^	0.242^**^	0.737^**^	1	
ATT	0.248^**^	0.391^**^	0.347^**^	0.273^**^	0.543^**^	0.699^**^	1

** *Significant at the level: 0.01 error*.

### The Effects of Independent Variables on the Dependent Variable

**Figure 3:**
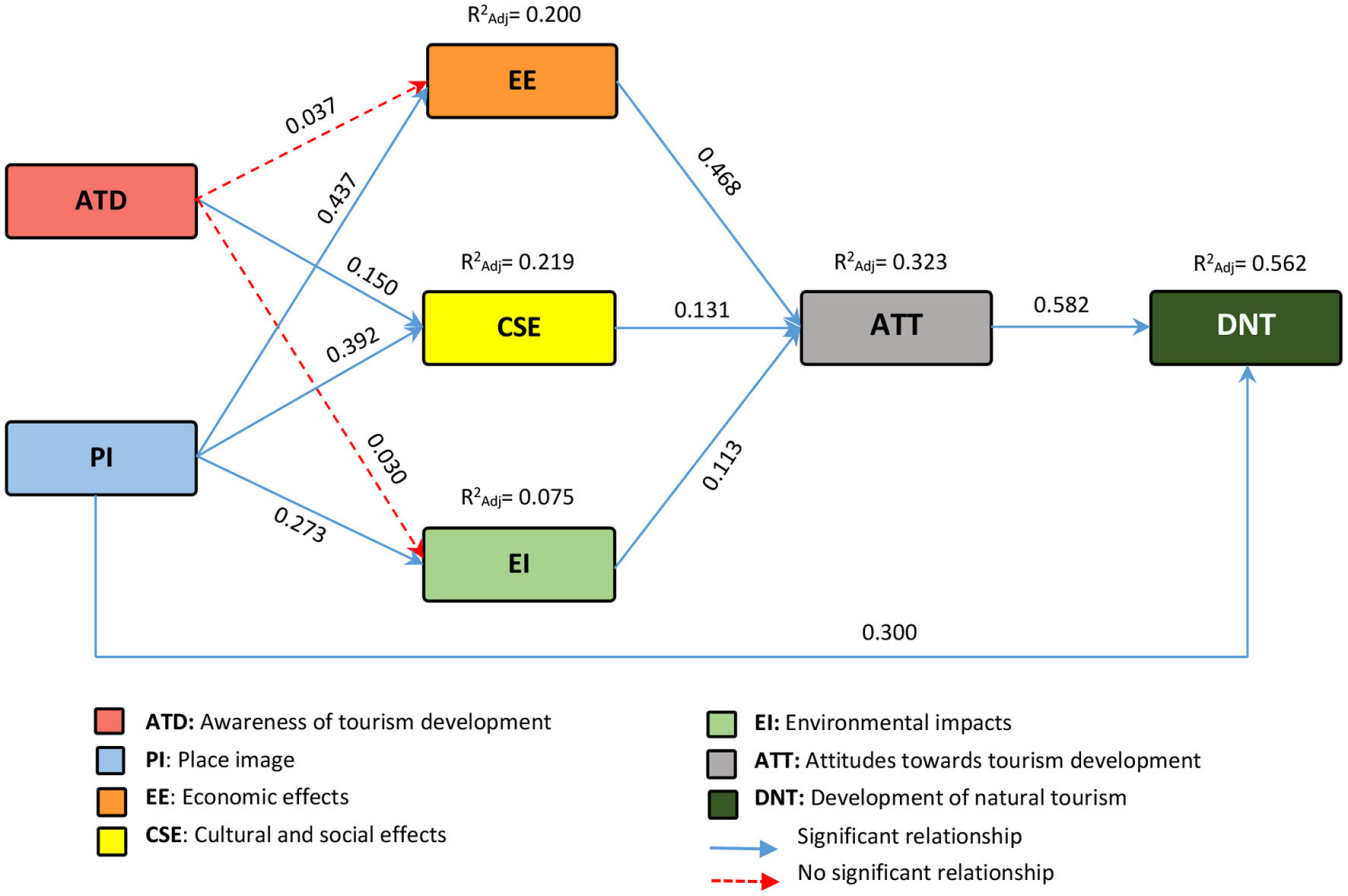
The causal research framework.

Path analysis technique was used to investigate the relationships between the variables (According the Figure 1). The findings of multiple regression analysis revealed that the framework could predict 56% of DNT variance changes, 32% of ATT variance changes, 20% of EE variance changes, 21% of CSE variance changes, and 0.7% of EI variance changes. In order to facilitate the calculations related to path analysis, first the direct effects on the dependent variables were calculated and in the next step, the ENTER multiple regression analysis method was used (Table 4; Figure 3).

**Table 4:** Direct effects on DNT, PI, and ATT.

**Direct effects on**	**Independent variables**	**B**	**β**	***t***	**t Sig**.
DNT	Constant	−1.858	—	−1.902	0.058
	PI	0.224	0.300	6.796	0.000
	ATT	0.514	0.582	13.173	0.000
	**F Sig = 0.000**	**F = 171.134**	${\text{R}}_{\text{Adj}}^{2}$ **= 0.562**	**R**^**2**^ **= 0.565**	**R = 0.752**
ATT	Constant	10.848	—	7.939	0.000
	EE	0.599	0.468	8.852	0.000
	CSE	0.214	0.131	2.289	0.023
	EI	0.231	0.113	2.066	0.040
	**F Sig . = 0.000**	**F = 34.159**	${\text{R}}_{\text{Adj}}^{2}$ **= 0.323**	**R**^**2**^ **= 0.331**	**R = 0.575**
EE	Constant	7.719	—	8.049	0.000
	**ATD**	0.038	0.037	0.619	0.537
	PI	0.315	0.437	7.225	0.000
	**F Sig**. **= 0.001**	**F = 21.91**	${\text{R}}_{\text{Adj}}^{2}$ **0.200**	**R**^**2**^ **=0.206**	**R = 0.454**
CSE	Constant	3.981	—	5.346	0.000
	**ATD**	0.118	0.150	2.502	0.013
	PI	0.222	0.392	6.549	0.000
	**F Sig**. **= 0.000**	**F= 38.121**	${\text{R}}_{\text{Adj}}^{2}$ **0.219**	**R**^**2**^ **= 0.225**	**R= 0.474**
EI	Constant	7.850	—	12.129	0.000
	**ATD**	0.019	0.030	0.469	0.640
	PI	0.123	0.273	4.197	0.000
	**F Sig**. **= 0.000**	**F = 11.816**	**R = 0.075**	**R**^**2**^ **= 0.082**	**R = 0.287**

The study of direct effects on DNT showed that the variables PI (*p* < 0.000; β = 0.300), ATT (*p* < 0.000; β = 0.582) had a positive and significant effect on DNT.

In the next step, the direct effects of independent variables on ATT were investigated and the findings showed that EE (*p* < 0.000; β = 0.468), CSE (*p* < 0.023; β = 0.131), and EI (*p* < 0.040; β = 0.113) had significant effects on ATT.

At the third stage, the direct effects of independent variables on EE were investigated and the findings showed that PI (*p* < 0.000; β = 0.437) had a significant effect on EE. Moreover, the effect of Awareness (n.s., *t* = 0.537) on the dependent variable was not significant.

In the fourth step, the direct effects of independent variables on CSE were investigated and the results showed that Awareness (*p* < 0.000; β = 0.150), PI (*p* < 0.000; β = 0.392) had significant effects on CSE.

At the fifth step, the direct effects of independent variables on EI were investigated and the results showed that PI (*p* < 0.000; β = 0.273) had a significant effect on EI. Also, the effect of Awareness variables (n.s., *t* = 0.640) on the dependent variable was not significant.

Finally, according to the theoretical framework, the effect of PI (*p* < 0.000; β = 0.300) and ATT (*p* < 0.000; β = 0.582) on DNT was investigated. The findings revealed a positive and significant effect of all three variables on DNT.

### Indirect and Total Effects on DNT

In Table 5, the causal effect (sum of indirect and direct effects) and the total causal effect affecting DNT were calculated. Indirect effects on DNT (according to theoretical framework) include indirect effects of ATD [(0.037 × 0.468 × 0.582) + (0.140 × 0.131 × 0.582) + (0.030 × 0.113 × 0.582) = 0.023], indirect effects of PI [(0.437 × 0.468 × 0.582) + (0.392 × 0.131 × 0.582) + (0.273 × 0.113 × 0.582) = 0.166], indirect effects of EE [(0.468 × 0.582) = 0.272], indirect effects of CSE [(0.113 × 0.582) = 0.076], and indirect effects of EI [(0.113 × 0.582) = 0.065]. As can be seen in Table 4 and Figure 3, in the path analysis performed, all paths (except the effects of ATD on EE and EI) conform to the theoretical framework (SET). In Table 3, the value of t was not significant for these two variables and on the other hand, the amount of β calculated for them was <0.05 (60); therefore, these two paths did not conform to Figure 1.

**Table 5:** Indirect and direct effects on DNT.

**Variables**	**Direct effects**	**Indirect effects**	**Total causal effects**	**Correlation coefficients**	**Non causal effects**	**Compliance with theoretical framework**
**ATD**	—	0.023	0.023	0.293	0.270	×
PI	0.300	0.166	0.466	0.528	0.062	✓
EE	—	0.272	0.272	0.737	0.465	✓
CSE	—	0.076	0.076	0.402	0.326	✓
EI	—	0.065	0.065	0.242	0.177	✓
ATT	0.582	—	0.582	0.699	0.117	✓
✓	Match the theory					
×	Mismatch with the theory					

The results of Table 5 showed that the variable of ATT has the most causal effect (0.582) and CSE has the least causal effect on DNT. EE also had the most non-causal effect (0.465) and EE and ATT had the least non-causal effect (both 0.177) on DNT.

## Conclusion

The present study aimed to develop tourism using SET and PI in the field of DNT with an emphasis on tourists in Sistan region. This study showed that SET can be used to explain DNT because the amount of variance explained by DNT was significant (56%). Due to the impact of SCE on tourists' ATT, it is necessary to consider tourism as a tool for community development. The tourism capacity during COVID-19 should be used to improve the image of recreational services provided, observe health issues, and improve tourists and residents' support of rural tourism development by holding various events in the Sistan region. Given the direct impact of PI on the development of rural tourism during COVID-19, a set of measures should be taken to improve the organic image (image that tourists obtain through their communication with residents) and the induced image of place (image that is indirectly affected by political and financial support of promotional activities). Tourists should be aware of the positive features of the place, security, and health assurance in the place they travel to, which leads to the development of tourism.

The results showed that ATT has the most causal effect and CSE has the least causal effect on DNT. Therefore, it is possible to take a big step in promoting DNT by creating a suitable platform for ATT amplification. Also, the necessary grounds should be provided to strengthen the causal effect of CSE. Finding missing links in this field can be a source for researchers' efforts in this direction.

One of the important findings has been the lack of effect of awareness perception on economic and environmental effects and of course its effect on tourists' attitudes during COVID-19. This result means that the environmental and economic effects of tourism for tourists have not been understood and tangible enough to change their attitudes; these effects and further development of tourism in the region should be felt in the long run. Other reasons for this can be seen in the small ability of tourists to assess the environmental impact of less developed tourist areas. According to these results, it can be said that the study area is at a good level in terms of social, cultural and institutional capabilities. It is not in a good level in terms of environmental capacity, due to lack of natural resource management and non-compliance with environmental behaviors and environmental health, as well as in terms of economic capacity due to lack of coherent and rational economic plans based on tourism and lack of funding. As a result, the government and stakeholder organizations must consider the credit and financial as well as the infrastructure of the study area. In terms of environmental potential, the promotion of environmental behavior, environmental management, identification and introduction of unique natural areas should be on the agenda.

Although this study provides a kind of causal conceptualization in DNT analysis, it faced some limitations. One of the most important was the COVID-19 pandemic, which made data collection difficult. Also, this research was conducted with a quantitative approach. Undoubtedly, for better results, a more comprehensive result can be achieved by using qualitative research methods as well as mixed methods. In this regard, the following suggestions can be made for future research.

- Analyzing rural tourism development in the post-Corona era;
- Designing an extension model of community-based ecotourism in rural areas and
- Pathology of rural tourism development in the pandemic of COVID-19: Application of grounded theory.

## Data Availability Statement

The raw data supporting the conclusions of this article will be made available by the authors, without undue reservation.

## Ethics Statement

Ethical review and approval was not required for the study on human participants in accordance with the local legislation and institutional requirements. Written informed consent for participation was not required for this study in accordance with the national legislation and the institutional requirements.

## Author Contributions

All authors listed have made a substantial, direct, and intellectual contribution to the work and approved it for publication.

## Conflict of Interest

The authors declare that the research was conducted in the absence of any commercial or financial relationships that could be construed as a potential conflict of interest.

## Publisher's Note

All claims expressed in this article are solely those of the authors and do not necessarily represent those of their affiliated organizations, or those of the publisher, the editors and the reviewers. Any product that may be evaluated in this article, or claim that may be made by its manufacturer, is not guaranteed or endorsed by the publisher.
